# "Synergistic Potential of Linagliptin and Resveratrol in Mitigating Type-II Diabetes-Induced Nephropathy": A Comprehensive Review

**DOI:** 10.2174/0115733998402561251202101133

**Published:** 2026-03-06

**Authors:** Prashant Ghimiray, Anand Vijayakumar P.R., Meghana G.R., Rimsha Jamil

**Affiliations:** 1 Department of Pharmacology, JSS College of Pharmacy, JSS Academy of Higher Education and Research, Ooty, Nilgiris, Tamil Nadu, India

**Keywords:** Diabetic Nephropathy, Linagliptin, Resveratrol, DPP-4 inhibitors, AMPK/Nrf2 pathway, type II diabetes mellitus

## Abstract

This narrative review aims to elucidate the significance of the synergistic effects of linagliptin and resveratrol and also explores their individual mechanisms of action in diabetic complications such as nephropathy. A systematic literature analysis examined the synergistic potential of resveratrol and linagliptin in diabetic nephropathy. A critical review of their antioxidant, anti-inflammatory, and anti-fibrotic mechanisms was conducted. Various studies revealed that linagliptin improves renal outcomes through both incretin-dependent and independent pathways, reducing oxidative stress, inflammation, and AGE-RAGE axis activation. It also exhibits the capacity to reduce albuminuria and renal fibrosis in diabetic animal models. Resveratrol similarly demonstrates substantial renoprotective benefits through activation of the AMPK/Nrf2 pathway, mitigating oxidative stress and downregulating inflammatory markers such as tumor necrosis factor-alpha and interleukin-1 beta. The synergistic use of linagliptin and resveratrol may provide greater benefits than their individual effects, including improved glycaemic control, reduced renal oxidative stress, and decreased inflammatory and fibrotic markers, thereby offering superior protection against diabetic nephropathy.

## 1 INTRODUCTION

Type II diabetes mellitus (T2DM) is a chronic metabolic disorder characterized by insulin resistance and impaired insulin secretion. By 2030, the number of individuals with diabetes mellitus is projected to reach 552 million, an increase from an estimated 366 million in 2011. Approximately 80% of individuals with type 2 diabetes reside in low- and middle-income countries. Diabetes caused approximately 4.6 million fatalities in 2011. Type 2 diabetes will continue to affect millions globally, with numbers expected to rise significantly by 2040 [1]. The incidence of type 2 diabetes varies geographically according to environmental and behavioural risk factors. T2DM is primarily associated with oxidative stress, which plays a crucial role in its pathogenesis and leads to complications including retinal degeneration, nerve damage, renal disease, and cardiovascular and peripheral artery disease [2].

One of the chronic kidney diseases in patients beginning renal replacement treatment is diabetic nephropathy, which is also linked to a higher risk of cardiovascular death. The traditional definition of diabetic nephropathy is proteinuria of ≥0.5 g/24 hours, a stage also referred to as proteinuria, macroalbuminuria, clinical nephropathy, or overt nephropathy [3]. About one-third of diabetic patients develop diabetic nephropathy, a serious consequence of both type 1 and type 2 diabetes that can result in end-stage renal disease (ESRD). Estimates indicate that the worldwide prevalence of adult diabetes may rise from 6.4% in 2010 to 7.7% in 2030, with China and India accounting for 154 million cases. Diabetic kidney disease (DKD), which develops in approximately one-third of individuals with type II diabetes, is expected to become more widespread given the notable global rise in type II diabetes over the next two decades. Microalbuminuria affects approximately 39% of diabetics worldwide.

Beyond the associated macrovascular effects, diabetic renal disease imposes a major economic burden for ESRD therapy. The projected yearly cost of treating ESRD in US type-II diabetics increased from USD 16.74 billion in 1998 to USD 39.35 billion in 2010. In affluent countries, diabetes causes ESRD to arise not only among Native Americans but also among African Americans and Indigenous Australians. Research indicates that these ethnic groups, particularly in countries most affected by diabetic nephropathy, face substantial challenges in meeting treatment costs [4].

Diabetic nephropathy is the primary cause of end-stage renal disease globally and creates significant health issues leading to death among people with diabetes mellitus. The major diagnostic sign of DKD is albuminuria, which contributes to cardiovascular complications and progressive kidney deterioration. The development of DKD depends on changes in renal hemodynamics, oxidative stress, inflammation, hypoxia, and elevated activity of the renin-angiotensin-aldosterone system, with kidney fibrosis playing a pivotal role [5].

Diabetic nephropathy is a complex and progressive condition involving a variety of cells, molecules, and factors. While the primary cause remains elusive, several mechanisms have been proposed, including hyperglycaemia, which leads to hyperfiltration and renal damage, as well as oxidative stress, inflammation, and fibrosis. Current therapeutic strategies include renin-angiotensin system (RAS) blockade, with renin inhibitors such as aliskiren showing potential in reducing proteinuria and albuminuria. However, research indicates that the overall effect on renal function, assessed using serum creatinine and estimated glomerular filtration rate, remains limited [6].

## 2 PATHOPHYSIOLOGY OF TYPE 2 DIABETES INDUCED NEPHROPATHY

A complex interplay between hemodynamic and metabolic variables is probably involved in the etiology of diabetic nephropathy. Advanced glycation end products, along with their receptors, represent glucose-dependent processes that function as metabolic variables. The renin-angiotensin system, together with other vasoactive hormones, represents hemodynamic factors. Protein kinase C and nuclear factor kappa-light-chain-enhancer of activated B cells, along with other signalling and molecular pathways, connect these metabolic and hemodynamic variables during adverse outcomes. Both the tubulointerstitial and the glomerulus, especially the podocytes, suffer pathological harm as a result of these contributory components [6].

### 2.1 Metabolic Factors

Increased ROS generation and activation of MAPK, JAK, and NF-κB signalling result from elevated blood glucose levels activating several pathways, including hexosamine, polyol, AGE, and PKC. Through direct ROS-mediated cellular damage, NF-κB-induced inflammatory cytokines, and MAPK-mediated extracellular matrix formation, these pathways contribute to inflammation and fibrosis [7].

Protein kinase C (PKC) is a key enzyme that regulates cell growth and function [8]. PKC serves as an intracellular signalling molecule capable of modulating diabetic complications, notably diabetic kidney disease (DKD). The PKC family encompasses multiple isoforms (α, βI, βII, γ, δ), each exhibiting distinct functions in the pathogenesis of DKD. PKC and nuclear factor kappa-light-chain-enhancer of activated B cells, along with other signalling and molecular pathways, connect metabolic and hemodynamic variables during adverse outcomes. The PKC signalling pathway is implicated in the progression of diabetic kidney disease by inducing constriction of small renal blood vessels, thereby adversely affecting glomerular function. Scientific research has demonstrated that inhibiting PKC activity reduces excessive vasoconstriction, decreases renal perfusion pressure, and mitigates glomerulosclerosis and impaired filtration rate. These findings highlight the critical role of PKC activation in the etiology of diabetic kidney disease [7].

High levels of advanced glycation end products (AGEs) are present in diabetic patients, in addition to being naturally formed during normal physiological processes [9]. AGEs arise from nonenzymatic interactions between glucose and amino groups in proteins, lipids, and nucleic acids [10]. In diabetes mellitus, elevated circulating AGEs activate the receptor for AGEs (RAGE), which triggers downstream signal transduction through MAPK, p38, SAPK/JNK, ERK1/2, and JAK/STAT/p38 pathways. This interaction sustains transcription factors such as NF-κB, STAT3, HIF-1α, and AP-1, contributing to oxidative stress and inflammation. Modulating the AGE-RAGE signalling axis is a potential therapeutic approach for diabetic nephropathy [7].

The hexosamine pathway is a proposed mechanism underlying glucose-induced toxicity and microvascular complications in diabetes mellitus. Its terminal phase involves the O-GlcNAc modification of proteins mediated by the pathway's principal product, UDP-N-acetylglucosamine. Although the role of the hexosamine pathway in extracellular matrix accumulation in diabetic nephropathy is well established, its influence on renal cell cycle perturbation and hypertrophy has received comparatively limited investigation [11]. Elevated glucose levels contribute to diabetic complications by increasing fructose-6-phosphate concentration, directing it towards the hexosamine biosynthetic pathway. Higher blood glucose stimulates cellular metabolic activities that generate cytokines such as TGF-β, ICAM-1, VCAM-1, TNF-α, CTGF, and plasminogen activator inhibitor-1 (PAI-1). These cytokines, in combination, act as mediators of various diabetes-related pathologies [7].

Hyperglycemia also activates the polyol pathway, resulting in sorbitol and fructose accumulation in renal cells, which triggers oxidative stress, inflammation, and AGE formation [12]. Research in diabetic murine models indicates that inhibition of aldose reductase (AR) can significantly mitigate early indicators of DKD, including reduced extracellular matrix accumulation, glomerular enlargement, and increased albumin excretion. However, AR inhibitors have shown limited effectiveness in human subjects. Hyperglycaemia-induced activation of the polyol pathway, leading to elevated tissue fructose levels, along with mitochondrial redox imbalance and NAD+ depletion *via* mechanisms involving poly ADP-ribosylation, sirtuins, CD38, and NAD kinase, are pivotal factors in the onset of diabetic renal complications. These findings elucidate critical metabolic disruptions in the pathogenesis of DKD and highlight potential therapeutic targets for this severe diabetic complication [7].

### 2.2 Hemodynamic Factors

Haemodynamic factors include both a rise in systemic and intraglomerular pressure and activation of vasoactive hormone systems, including the renin-angiotensin-aldosterone system (RAAS) [13]. These determinants contribute to the elevation of glomerular filtration rate and renal plasma flow during the initial stages of diabetes mellitus [14].

Early diabetic nephropathy leads to glomerular hyperfiltration and hyperperfusion due to afferent arteriole dilatation and reduced arteriolar resistance. This results in increased transcapillary hydraulic gradient and glomerular capillary pressure. Haemodynamic changes reflect the failure of autoregulatory systems, which normally induce preglomerular vasoconstriction when perfusion pressure rises. Prostanoids, nitric oxide, atrial natriuretic factor, growth factors, and angiotensin II have been associated with glomerular haemodynamic dysregulation. Increased intraglomerular pressure may cause mesangial matrix overproduction and thickening of the glomerular basement membrane, resulting in glomerulosclerosis and renal dysfunction in diabetic nephropathy [15].

Recent findings indicate that tubular processes substantially contribute to intraglomerular hypertension in early diabetes. Increased glucose transport in the proximal tubule enhances glucose and salt reabsorption, reducing salt delivery to the distal nephron and altering tubuloglomerular feedback, thereby affecting arteriole dilation and constriction. Higher SGLT2-mediated sodium chloride transport decreases macula densa sodium delivery, leading to reduced tubuloglomerular feedback control of afferent arteriole diameter and efferent arteriole angiotensin II-mediated contraction. Insulin also increases proximal tubule salt and glucose transport while decreasing afferent arteriolar tone, promoting hyperfiltration through both direct and indirect mechanisms. These actions elevate glomerular perfusion pressure and hyperfiltration, accelerating diabetic kidney disease [7].

Diabetic nephropathy is further promoted by RAAS overactivity. This system produces angiotensin II, which induces vasoconstriction, aldosterone release, cellular proliferation, fibrosis, thrombosis, inflammation, and oxidative stress [16]. The balance of RAAS components protects against the development of diabetic nephropathy. Genetic studies on DN within RAAS have focused on polymorphisms such as AGT M235T, ACE I/D, and AT1R A1166C. Numerous studies suggest that AGT M235T does not influence the risk of diabetic nephropathy. A distinctive relationship exists between ACE I/D polymorphism and DN, with ethnicity and gender affecting susceptibility; Southeast Asian females show increased risk. Substantial evidence also supports a correlation between diabetic nephropathy and the AT1R A1166C polymorphism, particularly in type 2 diabetes, according to association studies and meta-analyses [15-20]. The role of less-studied RAAS variants, including renin, AT2R, and aldosterone synthase, in DN development remains to be clarified. Current knowledge of specific RAAS gene variants may guide diabetes prevention, treatment, and management (Rahimi, 2016). RAAS blockade has been shown to improve kidney outcomes, primarily based on placebo-controlled clinical trials (Fig. **1**) [17].

## 3 DPP-4 INHIBITORS: MECHANISM AND ROLE IN DIABETIC KIDNEY DISEASE

Dipeptidyl peptidase-4 (DPP-4) inhibitors are a newly recognized class of drugs prescribed in T2DM that potentiate insulin release after meals by protecting GLP-1 and GIP from enzymatic degradation (Thornberry and Kollwitz, 2009). The renoprotective effects of DPP-4 inhibitors in diabetic nephropathy are mediated through both incretin-dependent and -independent pathways [18]. They improve renal pathology by reducing oxidative stress and inflammation and by enhancing endothelial function [19]. These agents also inhibit AGE-RAGE pathways and modulate integrin-β and TGF-β-Smad signalling to reduce renal fibrosis [20]. In animal models, DPP-4 inhibitors exhibit anti-inflammatory effects, including reduced macrophage infiltration and suppressed NF-κB activity in renal tissues [21]. Moreover, DPP-4 inhibitors are associated with improvement in kidney fibrosis. Activation of DPP-4 influences TGF-β signalling and the progression of renal disease through modulation of microRNA-29s, suggesting that DPP-4 inhibition may represent a novel therapeutic strategy for the treatment of progressive renal diseases, including fibrosis [22].

### 3.1 DPP-4 Inhibitors: Brief Information

The first DPP-4 inhibitor licensed for the treatment of diabetes was sitagliptin in 2006, followed by vildagliptin, saxagliptin, linagliptin, and alogliptin. The European Medicines Agency has authorized vildagliptin for type 2 diabetes, whereas the FDA has not. Some class medications authorized in South Korea, Japan, and Argentina include gemigliptin, anagliptin, alogliptin, and teneligliptin. All of these agents are DPP-4 inhibitors with similar mechanisms of action [23].

Some studies have focused on the protective role of linagliptin in rats exposed to endotoxin-induced acute kidney injury. Linagliptin effectively reduces inflammatory cytokines such as TNF-α and IL-1β and decreases reactive oxygen species through activation of AMPK and upregulation of HO-1. This leads to reduced renal tubular damage, improved biochemical markers, and survival benefits under sepsis conditions. These studies indicate that linagliptin possesses anti-inflammatory and antioxidative effects, which may be crucial in the treatment of septic AKI, suggesting potential therapeutic applications for diabetic nephropathy beyond glycaemic control. This review primarily focuses on this particular pathway and its correlation with resveratrol [24] (Table **1**).

### 3.2 Clinical Data of DPP-4 Inhibitors, Including Linagliptin

DPP-4 inhibitors demonstrate promising therapeutic potential in renal protection, as evidenced by both preclinical and clinical studies, particularly in reducing albuminuria through dual mechanistic pathways. Their nephroprotective effects operate through GLP-1-dependent mechanisms, leveraging increased levels of intact incretin hormones, as well as through GLP-1-independent pathways. The ability to lower albuminuria, a key marker of kidney damage and a predictor of progressive renal disease, underscores the clinical significance of these agents. This dual mechanism of action distinguishes DPP-4 inhibitors from traditional renal protective therapies and suggests they may offer comprehensive nephroprotection. Validation of these effects through both preclinical and clinical studies strengthens the evidence supporting their potential role in kidney protection, extending their therapeutic utility beyond glycaemic control in diabetes management [34].

### 3.3 Details of Individual Drugs Under DPP-4 Inhibitors in Kidney Disease

#### 3.3.1 Alogliptin

The clinical effectiveness and safety of alogliptin in T2DM have been extensively studied. A meta-analysis reported significant reductions in HbA1c levels with both 12.5 mg and 25 mg doses during two-year treatment periods in metformin-treated patients who were not adequately controlled. Alogliptin requires dose adjustments based on renal function and has been shown to be effective and safe in haemodialysis patients over long durations. A 12-week study associated alogliptin treatment with improvements in glycaemic control and renal parameters, alongside enhanced endothelial function. In combination with ARBs, alogliptin demonstrated superior efficacy to sitagliptin in reducing albuminuria in T2DM patients with early nephropathy. The EXAMINE trial confirmed cardiovascular safety comparable to placebo in T2DM patients with recent acute coronary syndrome, with consistent eGFR changes and dialysis rates. Currently, the J-BRAND Registry is performing long-term outcome assessments in 20,000 T2DM patients by monitoring urinary albumin output and tracking microangiopathy development [34, 35].

#### 3.3.2 Saxagliptin

Randomized trials have demonstrated the effectiveness and safety of saxagliptin, both as monotherapy and in combination with other oral antidiabetic agents. In patients with moderate renal impairment (CrCl 30-50 mL/min), severe renal impairment (CrCl < 30 mL/min), and those on haemodialysis, it is recommended to reduce the saxagliptin dose to 2.5 mg once daily. Saxagliptin 2.5 mg significantly lowered HbA1c levels compared with placebo in patients with moderate to severe renal impairment but not in those with ESRD on haemodialysis. These results were replicated over 52 weeks in a trial of 170 T2DM patients with CrCl < 50 mL/min. Pooled safety data from 20 randomized trials showed comparable rates of adverse events, including renal function deterioration, between saxagliptin and comparator groups. The SAVOR-TIMI 53 trial involving 16,492 T2DM patients confirmed a neutral effect of saxagliptin on cardiovascular safety; however, hospitalization for heart failure was increased, primarily in patients with prior heart failure, elevated natriuretic peptides, or chronic kidney disease. Saxagliptin also reduced UACR in the overall population and in renal impairment subgroups [35, 37].

#### 3.3.3 Sitagliptin

Sitagliptin, the first approved DPP-4 inhibitor, has been shown to be effective and well-tolerated as monotherapy, in combination with other oral antidiabetic agents, and with insulin therapy. For patients with mild to moderate renal impairment (eGFR 30-50 mL/min/1.73 m^2^), the recommended dose is 50 mg per day. In patients with severe renal impairment undergoing haemodialysis, the dose should be reduced to 25 mg per day. Clinical studies demonstrate that sitagliptin is both safe and effective in improving glycaemic control in patients with stage 3-5 chronic kidney disease, including those on dialysis. A 54-week study of 65 patients with moderate to severe renal insufficiency showed that sitagliptin reduced HbA1c by 0.7%. Another 54-week trial comparing sitagliptin and glipizide in 426 patients demonstrated non-inferiority for HbA1c reduction, with sitagliptin providing higher rates of glycaemic target attainment without weight gain or hypoglycaemia. In 129 type 2 diabetes patients with ESRD on dialysis, sitagliptin achieved HbA1c levels comparable to glipizide [37, 34].

#### 3.3.4 Linagliptin

Clinical trials have demonstrated strong renal protective effects of linagliptin [38]. In a 24-week trial, linagliptin reduced albuminuria by 28% more than placebo, with effects observed at week 12 persisting independently of changes in glycaemic control. Small or large reductions in HbA1c produced similar decreases in ACR, supporting preclinical findings that linagliptin directly protects kidney function beyond blood glucose management [39]. A pooled analysis of 13 phase 3 trials involving 5,466 patients assessed a composite endpoint of new micro- or macroalbuminuria, worsening CKD, acute renal failure, and all-cause mortality. Linagliptin demonstrated superior outcomes versus placebo, primarily through reductions in micro- and macroalbuminuria, with patients under 65 benefiting most. The MARLINA-T2D trial confirmed that linagliptin lowers albuminuria in type 2 diabetics with renal impairment, while the ongoing CARMELINA trial is evaluating its long-term cardiovascular and renal effects. These studies support linagliptin’s role as a renoprotective agent (Fig. **2**, Table **2**) [39].

## 4 RESVERATROL: MECHANISM AND THERAPEUTIC POTENTIAL IN DIABETIC NEPHROPATHY

The natural organic compound resveratrol (RES), or 3,4,5-trihydroxy-stilbene, exists as a phytoalexin and non-flavonoid phytoalexin that belongs to the polyphenolic stilbene category [40]. Resveratrol was first isolated in 1940 by the Japanese scientist Michio Takaoka from *Veratrum grandiflorum* (white hellebore) [41]. In 1963, Nomomura isolated resveratrol from the Japanese knotweed (*Polygonum cuspidatum*), and in 1992, Siemann and Creasy identified resveratrol in wine [42]. The biological activities of resveratrol span multiple pharmacological functions, including anti-obesity, anti-diabetic, anti-cancer, anti-inflammatory, antioxidant, and cardiovascular protective effects (Figs. **3** and **4**) [43].

Resveratrol demonstrates anti-inflammatory, anti-tumoral, antidiabetic, and cardioprotective properties, particularly in the management of diabetes mellitus and its associated vascular complications, through improved nitric oxide-mediated vasodilation. Furthermore, resveratrol provides protection against several age-related ailments, including cardiovascular diseases, certain malignancies, arthritis, and cognitive decline, making it beneficial both as a therapeutic adjunct for chronic disease management and as a preventative measure in healthy individuals [44].

Resveratrol exhibits renoprotective effects against diabetic nephropathy *via* multiple critical pathways and mechanisms, including modulation of oxidative stress and advanced glycation end-products (AGEs), regulation of autophagy, attenuation of lipotoxicity, endoplasmic reticulum stress and inflammation, activation of the AMP-activated protein kinase (AMPK) pathway, and regulation of angiogenesis. Consequently, clinically significant therapeutic benefits in diabetic kidney disease can be achieved through the simultaneous activation of these pathways [45].

Hyperglycaemia activates many pathogenic pathways that lead to vascular complications in diabetes, including intracellular sorbitol synthesis, protein kinase C activation, mitochondrial superoxide production, and formation of AGEs. Together, these mechanisms generate oxidative stress, inflammation, and nitrosative stress, ultimately causing vascular damage. Resveratrol, owing to its potent anti-inflammatory and antioxidant properties, can interrupt these harmful pathways and protect against diabetic vascular complications by targeting multiple stages of this pathogenic cascade. Additionally, resveratrol improves glucose homeostasis, reduces insulin resistance, and protects pancreatic β-cells, largely through upregulation of AMPK and Sirtuin 1 expression and activity [43].

### 4.1 Antioxidant

Hyperglycaemia causes excessive generation of reactive oxygen species (ROS), which overwhelms the body’s antioxidant defenses, leading to oxidative stress and diabetic complications. This imbalance between ROS production and antioxidant capacity contributes to multiple diabetes-related pathologies [46]. Studies examining oxidative markers, along with GSH, CAT, and SOD assays, have demonstrated that resveratrol (RES) exhibits antioxidant effects in diabetic animals. Its protective mechanism against oxidative stress involves the activation of antioxidant enzymes that neutralize free radicals [47].

RES primarily scavenges superoxide radicals and enhances SOD mRNA expression in various diabetic mouse models. The upregulation of SOD mRNA is central to RES’s antioxidant activity. RES also increases SIRT1 expression in diabetic liver tissues, which possesses antioxidant properties. Under oxidative stress, SIRT1 promotes FOXO deacetylation, enhancing transcription of target genes, including SOD. Through the SIRT1-FOXO pathway, RES increases SOD expression and mitigates oxidative damage [48].

Evidence indicates that in diabetes, organs such as the testis, liver, and kidney exhibit NADPH oxidase as a principal source of ROS. Excessive ROS formation in diabetic individuals is stimulated by hyperglycaemia, elevated fatty acids, and advanced glycation end-products, all of which activate NADPH oxidase [49, 50].

### 4.2 AMPK/Nrf2 Pathway (Antioxidant Activity)

AMPK plays a crucial role in regulating the antioxidant activity of resveratrol (RES). In diabetic mice, RES treatment activates AMPK, leading to downregulation of NADPH oxidase and elevated SOD expression levels [47, 48].

Uncoupling protein 2 (UCP2) is located in the inner mitochondrial membrane and serves multiple protective functions in diabetes mellitus. It reduces ROS production and oxidative stress while enhancing cellular glutathione (GSH) levels. The effects of UCP2 vary across tissues: in adipose tissue and the kidneys, its overexpression mitigates diabetes-associated complications, whereas in pancreatic islets, it enhances cellular function. Through these coordinated activities, UCP2 acts as a key negative regulator of oxidative stress-related diabetic consequences [49, 50].

Reduction of oxidative and electrophilic stress also depends on nuclear factor erythroid-2-related factor 2 (Nrf2), which regulates cellular redox status and energy metabolism [51]. Recent studies demonstrate that RES combats oxidative stress by modulating the Nrf2 pathway. Nrf2 functions as an antioxidant transcription factor that controls the expression of antioxidant enzymes, including heme oxygenase-1 (HO-1), NADPH quinone oxidoreductase-1, and others, thereby protecting cells against redox stress damage [52].

### 4.3 Anti-inflammatory

Inflammation occurs in response to tissue injury and various diabetic pathogenic stresses and may contribute to the development of diabetes by inducing insulin resistance. Chronic diabetic complications can be exacerbated by inflammation, particularly in the context of hyperglycaemia [49]. Studies using both *in vitro* cell-based models and animal experiments have confirmed that resveratrol (RES) exhibits substantial anti-inflammatory properties. This effect is associated with reduced expression of proinflammatory cytokines, including IL-1β, IL-6, TNF-α, as well as iNOS and COX-2 [50].

Resveratrol mitigates diabetes and its complications primarily in animal models of diabetes mellitus or insulin resistance, induced by streptozotocin (STZ) injection or high-fat diets. Several studies have evaluated the anti-inflammatory effects of RES in liver, testicular, and renal tissues, highlighting its protective role against diabetes-related tissue damage [53, 54].

### 4.4 AMPK/Nrf2 (Anti-inflammatory Activity)

The anti-inflammatory mechanism of resveratrol (RES) relies primarily on the functional activation of the AMPK pathway [53]. When the phosphoinositide AMPK pathway is significantly disrupted, RES is less able to reduce the synthesis of pro-inflammatory cytokines, including COX-2 and iNOS, in diabetic tissues and cells. Excessive iNOS production generates high levels of nitric oxide, which can directly initiate and exacerbate diabetic complications. COX-2 primarily produces prostaglandins, which act as key mediators in the development of diabetic nephropathy [54].

The anti-inflammatory effects of RES are also strongly associated with the MAPK signalling pathways. Partial activation of MAPK occurs in response to inflammatory stimuli, and RES-mediated inactivation of MAPK signalling requires functional AMPK activity, as shown in macrophage studies [55].

Furthermore, the anti-inflammatory actions of RES are closely linked to the transcription factor Nrf2. Heme oxygenase-1 (HO-1) functions as an antioxidant enzyme whose expression is regulated by Nrf2 and stimulated by RES. RES therapy activates AMPK, which promotes Nrf2 nuclear translocation and suppresses pro-inflammatory cytokine production. Several studies indicate that HO-1 is involved not only in the anti-inflammatory effects of RES but also in its antioxidant protection. Reduction of HO-1 diminishes RES’s ability to suppress pro-inflammatory cytokine synthesis.

## 5 EFFECTIVENESS OF RESVERATROL IN DIABETES AND ITS COMPLICATIONS

The increasing prevalence of diabetes, its involvement in multiple signalling pathways, diverse aetiology, progressive course, and associated comorbidities underscore the need for multifunctional therapeutic agents. Red wine contains resveratrol, a natural polyphenol with significant potential in the treatment of diabetic kidney disease (DKD) through multiple mechanisms. Resveratrol exhibits antioxidative, anti-inflammatory, and antifibrotic effects, protecting against renal damage induced by oxidative stress. It interacts with several proteins, including PPARA, SHBG, AKR1B1, PPARG, IGF1R, MMP9, AKT1, and INSR, to deliver therapeutic benefits in DKD [56].

The primary mechanisms underlying the therapeutic effects of resveratrol include activation of NAD-dependent deacetylase sirtuin-1 (SIRT1) and AMP-activated protein kinase (AMPK), along with enhancement of intracellular antioxidant capacity. Preclinical studies indicate that combination therapy with these pathways may provide enhanced protection against diabetes, a noteworthy discovery. Consequently, validation of these findings in rigorously conducted clinical trials remains crucial for identifying new therapeutic strategies for the treatment of diabetes and its associated complications [57].

Resveratrol has been shown to mitigate the detrimental effects of diabetes in numerous *in vitro* and *in vivo* animal studies through distinct mechanisms [58]. Several interconnected pathways have been implicated in accelerating the onset of diabetes-related complications. Research demonstrates that resveratrol improves biochemical and clinical markers in animal models of diabetic nephropathy, including both type 1 and type 2 diabetes, as well as in models of diabetic neuropathy, diabetic retinopathy, diabetes-induced cardiovascular disease, and diabetes-induced liver injury [42] (Tables **3** and **4**).

## 6 SYNERGISTIC POTENTIAL OF LINAGLIPTIN AND RESVERATROL IN DIABETIC KIDNEY DISEASE

Recent research has examined the synergistic effects of DPP-4 inhibitors and natural compounds in type 2 diabetes therapy, with a focus on resveratrol. DPP-4 inhibitors enhance incretin levels, thereby lowering blood glucose and increasing insulin secretion [64]. This review primarily focuses on the combined effects of the DPP-4 inhibitor linagliptin and resveratrol (a phytoalexin) in high-fat diet and low-dose streptozotocin-induced type 2 diabetic male Sprague Dawley rats, highlighting their potential role in future therapies for diabetes-related complications, including diabetic neuropathy, diabetic nephropathy, and diabetic retinopathy.

Concurrent treatment with DPP-4 inhibitors and resveratrol demonstrated the anticipated therapeutic outcomes, including decreased blood glucose levels, sustained serum insulin concentrations, improved glucose sensitivity, and reduced insulin resistance. The combination also positively influenced insulin resistance, circulating lipids, glycaemic indices, oxidative stress, and inflammatory status [65]. Since resveratrol may alter the pharmacokinetics of certain DPP-4 inhibitors, dosage adjustments could be necessary [66].

These findings indicate that combining DPP-4 inhibitors with natural compounds such as resveratrol may offer novel therapeutic strategies for diabetes management, although further research is required. The combination of resveratrol and linagliptin is particularly justified, as linagliptin has been shown to restore or activate the AMPK pathway and HO-1, suppress pro-inflammatory cytokines such as TNF-α and IL-1β, mitigate ROS overproduction, and improve renal outcomes in rat models, effects similar to those observed with resveratrol (Fig. **5**) [24].

### 6.1 Resveratrol

The antioxidant activity of resveratrol (RES) appears to be strongly influenced by AMPK activation under specific conditions. Activation of AMPK leads to increased expression of superoxide dismutase (SOD), which contributes to the antioxidant effects observed in diabetic models treated with RES (Fig. **6**) [43].

### 6.2 Combined Hypothetical Pathway of Resveratrol and Linagliptin that has been Postulated

Based on literature reviews and collected data, resveratrol and linagliptin may represent an effective combination for the management of diabetes and diabetes-related complications, such as diabetic nephropathy. Resveratrol can interact with certain medications, specifically DPP-4 inhibitors like alogliptin (ALO), saxagliptin (SAX), and sitagliptin, by inhibiting cytochrome P450 enzymes responsible for drug metabolism. This inhibition can slow the breakdown of these drugs, potentially prolonging their effects and increasing the risk of side effects. However, no such interactions have been reported for linagliptin to date, suggesting that the combination with resveratrol may be safe for various diabetic complications [66].

Based on multiple literature reviews, we have proposed a hypothetical pathway that may play an effective role in modulating diabetic complications, as depicted in Fig. (**7**).

## CONCLUSION

The combination of resveratrol, a phytoalexin with antioxidant and anti-inflammatory properties, and linagliptin, a DPP-4 inhibitor with renoprotective potential, represents a promising therapeutic strategy for the treatment of diabetes-related complications, such as diabetic nephropathy. Preclinical studies have demonstrated the synergistic effects of these agents, including reductions in oxidative stress, inflammation, and fibrosis, alongside improvements in glycaemic control and renal function. The unique pharmacological profiles of resveratrol and linagliptin, particularly their ability to modulate key pathways such as AMPK, Nrf2, and the AGE-RAGE axis, highlight their potential for combined application.

To confirm these effects, determine optimal dosing, and evaluate the long-term safety and efficacy of this combination, future studies, including well-designed clinical trials, are essential. Through such an integrated approach, diabetic nephropathy and its associated complications may be managed more effectively, offering innovative and comprehensive therapeutic options to address unmet needs in current diabetes treatment strategies.

## Figures and Tables

**Fig. (1):**
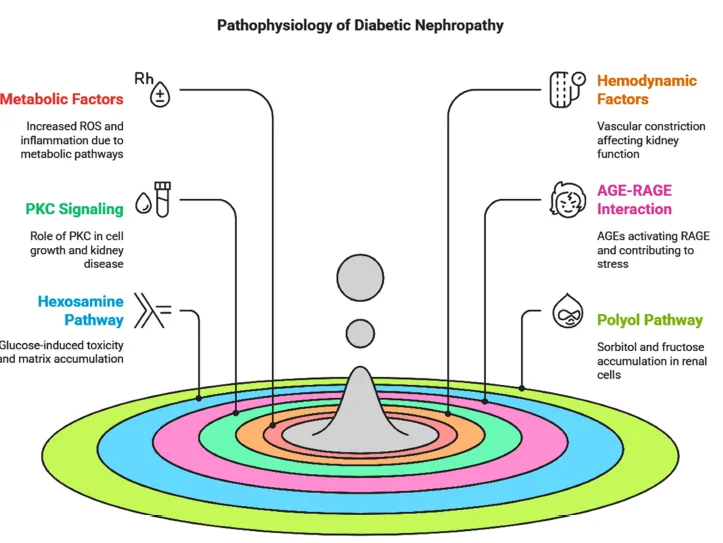
Metabolic and hemodynamic factors.

**Fig. (2):**
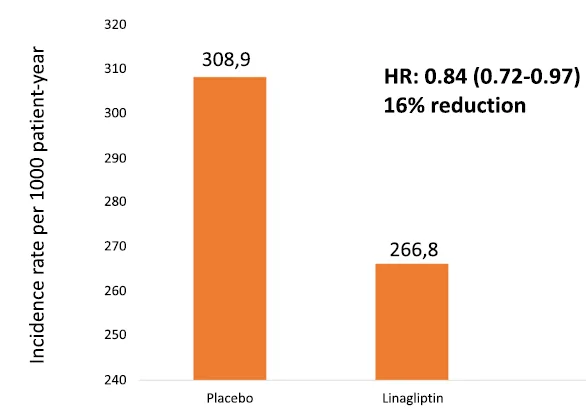
The Graph represents the reduction of risk in the group of linagliptin as compared to placebo.

**Fig. (3):**
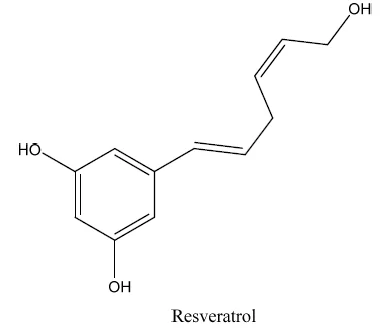
Molecular structure of resveratrol.

**Fig. (4):**
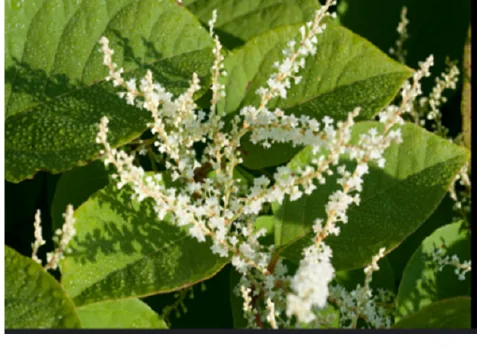
Polygonum cuspidatum.

**Fig. (5):**
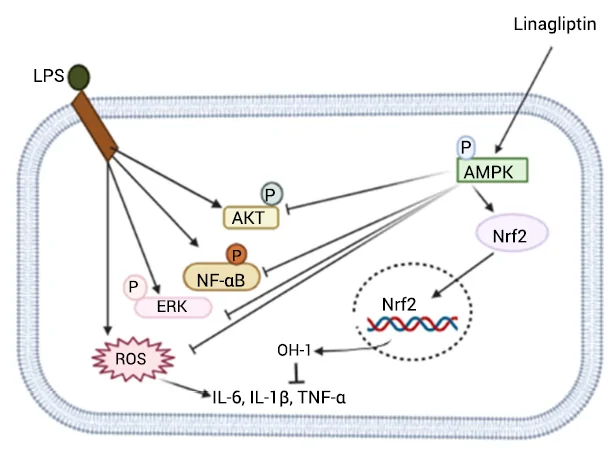
Postulated pathway of Linagliptin in Endotoxin induced kidney disease.

**Fig. (6):**
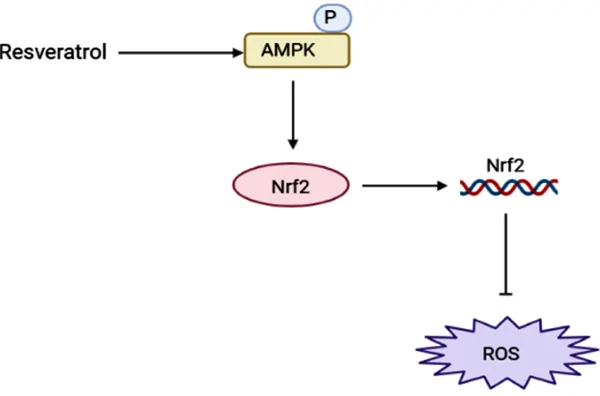
Effect of Resveratrol in the AMPK/Nrf2 pathway.

**Fig. (7):**
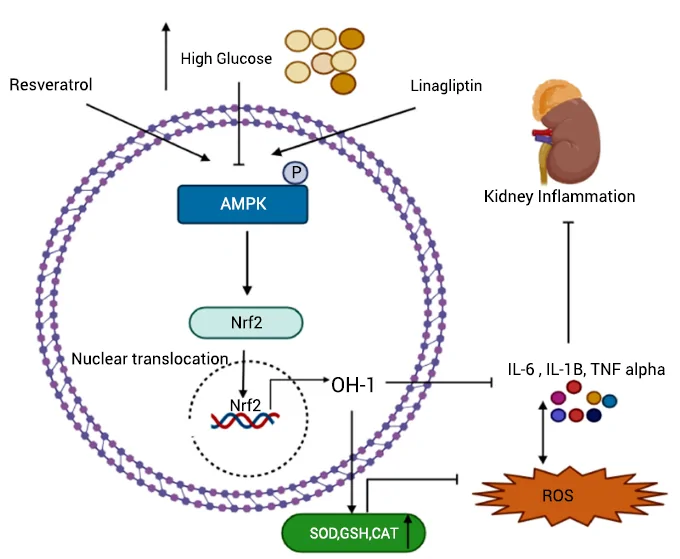
Hypothetical pathway of combine effect of Resveratrol and DPP-4 inhibitor.

**Table 1:** Preclinical data of DPP-4 inhibitor (Linagliptin) in Diabetic Nephropathy.

**Animal Models**	**The Effect of Linagliptin**	**Total Sample Size**	**References**
**Diabetic** Streptozotocin-induced diabetic, eNOS knockout C57BL/6J mouse	Reduced Glomerulosclerosis. Reduced Renal. Oxidative Stress (in Monotherapy). Reduced Albuminuria (combined with telmisartan).	60	[25]
Streptozotocin-diabetic SD rats	Reduced Albumin level in urine. Reduced advanced glycated end product and Receptor Advanced Glycated End product. Reduced Oxidative.stress (Renal). Reduced Infiltration. Of. Lymphocyte of the glomerulus.	30	[26]
db/db,C57BL6 mouse	Reduced Albumin in Urine. Reduced Glomerulosclerosis. Reduced Tubulointerstitial fibrosis. Reduced Podocyte damage.	36	[27]
STZ-diabetic CD-1 mouse	Reduced Glomerulosclerosis. Reduced Tubulointerstitial fibrosis. Reduced Albuminuria.	20	[28]
Glp 1r^+/+^ and Glp 1r^-/-^ diabetes-prone Akita mice	Reduced Albuminuria (only in Glp1r^+/+^ mice). Reduced Glomerulosclerosis. Reduced Periglomerular fibrosis. Reduced loss of Podocyte Reduced Oxidative stress (Renal).	27	[29]
STZ-diabetic Sprague-Dawley rat	Reduced Albuminuria. Reduced Renal Oxidative Stress.	-	-
**Non-diabetic** Wistar rats with 2-kidney-1-clip surgery, Zucker obese rats	Reduced Oxidative Stress. Reduced Loss of slit pore diaphragm, podocyte effacement, and glomerular endothelial fenestrae. Reduced Renal DPP-4 activity without altering renal DPP-4 protein content. Reduced Proteinuria. Reduced Kidney tissue Dipeptyl Peptidase 4 Inhibitor activity. Reduced Active GLP-1 in.plasma. Reduced SDF-1α in the kidney and plasma	56 20	[30, 31]
Wister and Sprague.Dawley rat 5/6 nephrectomy	Reduced Interstitial.Fibrosis. Reduced Glomerular, Hypertrophy. Reduced Albumin in urine.	40	[32]
C57BL/6 mice are injected with free fatty acid-bound albumin peritoneally.	Reduced Tubular. Inflammation, fibrosis, and apoptosis. Reduced Tubular injury.	17	[33]

**Table 2:** Risk involved in two groups of patients.

Renel events	306	448
Time at risk, years	991	1679
Patients, n	1961	3505

Microalbuminuria, macroalbuminuria, CKD (s CR>2.8 mg/dl), worsening of CKD (loss e GFR >50% of baseline, acute renal failure and death from any cause.

**Table 3:** *In-vitro* reports of resveratrol in diabetic nephropathy.

**Description**	**Outcome**	**Significance**	**References**
In-vitro: The effects of RES (5-20 µmol/L were assessed against high-glucose-induced EMT changes in renal tubular epithelial cells.	By inhibiting ERK1/2 and downregulating NOX1 and NOX4, resveratrol reduced intracellular ROS and prevented high-glucose-induced EMT.	The cell resistance of Resveratrol stands strong against epithelial-mesenchymal transition effects caused by high glucose in tubular cells. (EMT) in renal tubular cells through obsolete inhibition of NOX/ROS/ERK1/2 pathway.	[57]
In-vitro: The combination of RES at 10 µmol/L concentration showed effects on both high-glucose-modelled mesangial cell growth, along with fibronectin protein expression levels.	Through activation of NAD(P)H oxidase activity and blockade of JNK and NF-KB activation, along with reduced ROS production, resveratrol prevented high-glucose-induced cell division and fibronectin synthesis in mesangial cells.	Research indicates resveratrol presents therapeutic potential to treat diabetic nephropathy through its application on the JNK/NF-Kb/NOX/ROS pathway.	[46]
Researchers evaluated RES at 10 µmol/L as an *in vitro* treatment for high-glucose-induced oxidative damage in rat mesangial cells.	Treatment with Resveratrol prior to exposure enhanced ROS production while simultaneously increasing mitochondrial superoxide production and Mn SOD activity, as well as mesangial cell mitochondrial complex III activity, which drives mitochondrial oxidative stress.	Cells under glucose conditions experience reduced oxidative stress together with mitochondrial protection when SIRT1 is activated by resveratrol.	[58]
In-vitro: Under cellular conditions, human renal epithelial cells exposed to high blood glucose concentration showed changes in response to resveratrol (25 µmol/L) treatment.	RES reduced oxidative stress and increased deacetylase activity of SIRT1 while lowering FOXO3a expression through acetylation, but the inhibitor blocked both its protection from oxidative stress and its beneficial effects.	The activity of deacetylase SIRT1 is strengthened through resveratrol administration to stop oxidative stress damage of renal tubules with elevated blood glucose levels.	[59]
*in vitro*: Resveratrol (10 µmol/L) affects high hyperglycaemia-driven oxidative stress and podocyte cell death.	Res increased SIRT1, PGC-1α, NRF1, and mitochondria transcription factor A expression, respiratory chain complexes I and III activity, and mitochondrial membrane Potential, while reducing reactive oxygen species and cell death caused by high glucose levels.	Resveratrol mitigates oxidative damage and death in podocytes induced by severe hyperglycaemia *via* mitochondrial protection facilitated by SIRT1/PGC-1α.	[60]

**Table 4:** *In-vivo* reports of resveratrol in diabetic nephropathy.

**Animal models**	**The Effect of Resveratrol**		**Total Sample Size**	**Reference**
*in vivo*: Resveratrol (5 and 10 mg/(kg-d)) effects on renal function and oxidative stress in diabetic rats.	Resveratrol reduced signs of pre-diabetic nephropathy, such as decreased creatinine and urea clearance, proteinuria, and oxidative stress, which is marked by increased lipid peroxidation and reduced antioxidant enzyme activity.	This research underscores the significance of resveratrol’s antioxidant ability in mitigating renal failure in diabetic nephropathy.	28	[61]
*in vivo*: Resveratrol (0.3% with non-purified food) on diabetic nephropathic db/db mice.	In db/db rats, resveratrol reduced kidney mitochondrial oxidative stress, biogenesis, and urine albumin excretion.	By normalising Mn SOD activity, resveratrol protects against diabetic nephropathy by increasing oxidative stress.	73	[50]
*in vivo*: The administration of Resveratrol at increasing concentrations (1,5,10 mg/Kg-d) lowers both renal RAGE and Oxidative Stress levels in diabetic rats.	After RES treatment, rats had higher insulin and antioxidant levels and lower plasma glucose, malondialdehyde, and RAGE.	Resveratrol reduces oxidative stress and RAGE expression to protect the Kidneys in diabetes.	40	[62]
*in vivo*: The administration of RES at increasing concentrations (1,5,10 mg/kg-d) lowers both renal RAGE and oxidative stress levels in diabetic.rats.	The administration of resveratrol to diabetic rats produced diminished levels of AGE accumulation and oxidative stress, together with decreased DNA damage. Studies show that RES minimizes both tubular atrophy and mesangial extension or contraction while preventing diffuse glomerulonephritis and fibrosis.	Current studies indicate RES effectively minimizes both oxidative damage and glycation while preventing apoptosis in order to delay diabetic nephropathy progression.	40	[63]

## LIST OF ABBREVIATIONS

AGE: Advanced Glycation End-products
AMPK: AMP-Activated Protein Kinase
AR: Aldose Reductase
CKD: Chronic Kidney Disease
COX2: Cyclooxygenase-2
CTGF: Connective Tissue Growth Factor
DPP-4: Dipeptidyl Peptidase-4
DKD: Diabetic Kidney Disease
DN: Diabetic Nephropathy
ESRD: End-Stage Renal Disease
FOXO: Forkhead Box Protein O
GIP: Glucose-Dependent Insulinotropic Polypeptide
GLP-1: Glucagon-Like Peptide-1
GSH: Glutathione
HbA1c: Glycated Hemoglobin
HO-1: Heme Oxygenase-1
IL-1β: Interleukin-1 Beta
IL-6: Interleukin-6
iNOS: Inducible Nitric Oxide Synthase
JAK: Janus Kinase
MAPK: Mitogen-Activated Protein Kinase
Mn SOD: Manganese Superoxide Dismutase
mRNA: Messenger Ribonucleic Acid
NADPH: Nicotinamide Adenine Dinucleotide Phosphate
NF-κB: Nuclear Factor Kappa-Light-Chain-Enhancer of Activated B Cells
Nrf2: Nuclear Factor Erythroid 2-Related Factor 2
PAI-1: Plasminogen Activator Inhibitor-1
PKC: Protein Kinase C
RAAS: Renin-Angiotensin-Aldosterone System
RAGE: Receptor for Advanced Glycation End-products
RES: Resveratrol
ROS: Reactive Oxygen Species
SGLT2: Sodium-Glucose Cotransporter 2
SIRT1: Sirtuin 1
STZ: Streptozotocin
TGF-β: Transforming Growth Factor-Beta
TNF-α: Tumor Necrosis Factor-Alpha
T2DM: Type 2 Diabetes Mellitus
UCP2: Uncoupling Protein 2
VCAM-1: Vascular Cell Adhesion Molecule-1
